# Minimally Invasive Approach for Complete Resection of a Cervical Intramedullary Tumor via a Dorsal Root Entry Zone Using Fixed Tubular Retractor

**DOI:** 10.7759/cureus.28457

**Published:** 2022-08-26

**Authors:** Bilal Tarabay, Antoine Gennari, Ghassan Boubez, Zhi Wang, Daniel Shedid, Sung-Joo Yuh

**Affiliations:** 1 Neurological Surgery, Centre Hospitalier de l'Université de Montréal (CHUM), Montreal, CAN; 2 Orthopaedic Surgery, Centre Hospitalier de l'Université de Montréal (CHUM), Montreal, CAN; 3 Neurosurgery, Centre Hospitalier de l'Université de Montréal (CHUM), Montreal, CAN

**Keywords:** minimally-invasive spine, intramedullary tumors, intramedullary ependymoma, spinal cord tumor surgery, minimally invasive surgical procedures

## Abstract

We describe the surgical aspects of the resection of a large 2cm intramedullary ependymoma at the C6-7 level associated with an extensive syrinx using a unilateral minimally invasive approach through a fixed tubular retractor. A gross total resection was achieved. Total operative time was 5 hours. Estimated blood loss was less than 100 cc. Postoperative evolution was favorable, with the improvement of the patient’s neurological status. There was no cerebrospinal fluid (CSF) fistula. Hospital stay was four days. All narcotics were stopped on day 1 after surgery. Post-operative MRI showed no residual tumor. At the six-month follow-up, there was continued improvement in his neurological status. Scoliosis films did not reveal any cervicothoracic kyphosis.

## Introduction

Intramedullary spinal cord (IMSC) tumors account for less than 5% of all spinal tumors. Of these, ependymomas and astrocytomas are the two most common types found in adults [1]. Surgical resection of these IMSCs is technically challenging and is associated with high risks of neurological deterioration [2]. Historically, the preferred treatment has mostly been conservative, consisting of a diagnostic biopsy followed by radiotherapy if warranted [3]. Literature suggested that patient survival, local recurrence rates and overall prognosis are all heavily dependent on the extent of microsurgical resection. Publications from the early 1980s by Leonard Malis [3] and Bennett Stein [4] described the microsurgical techniques for resection of intramedullary tumors. With the advent of MRI scanning and intraoperative neuro-monitoring, a more aggressive microsurgical resection of intramedullary tumors to achieve gross total resection (GTR) while preserving neurological functions is considered the favored approach [2].

Because of the high incidence of spinal deformities following laminectomies for resection of intramedullary tumors [5], many authors suggested alternative techniques to preserve the posterior midline ligaments, especially the interspinous ligament, such as laminoplasty and unilateral approaches [6], with reported good results. After exposure of the dura, a midline dural opening and retraction are performed, allowing the identification of the median sulcus of the spinal cord. Depending on its location, the tumor is exposed with a posterior midline myelotomy or through the dorsal root entry zone [7].

The use of minimally invasive surgery (MIS) approaches allows for a similar exposure while preserving the paraspinal muscle attachments and the posterior midline ligaments. MIS techniques for spinal intradural extramedullary tumors have been reported in many case series with satisfactory results [8-10]. They have been shown to achieve similar gross total resection rates when compared to open surgeries, all while associated with a decreased incidence of cerebrospinal fluid (CSF) leak, reduced post-operative pain and a shorter hospital stay [10].

Here we report a clinical case of complete resection of a large cervical intramedullary tumor associated with an extensive syrinx using a minimally invasive surgical approach via a dorsal root entry zone (DREZ), with a fixed tubular retractor. We present technical nuances for such minimally invasive approach, and a short-term neurological and radiographic finding.

## Case presentation

We report the case of a 42-year-old patient presenting to the outpatient neurosurgical clinic with worsening gait imbalance, prehension difficulties and bilateral distal hand weakness over the past six months. There was weakness noted in his intrinsics and flexor digitorum profundus bilaterally, which was graded at 2/5. There was mild spasticity noted as well in his lower extremities. His modified McCormick grade was IV.

**Figure 1:**
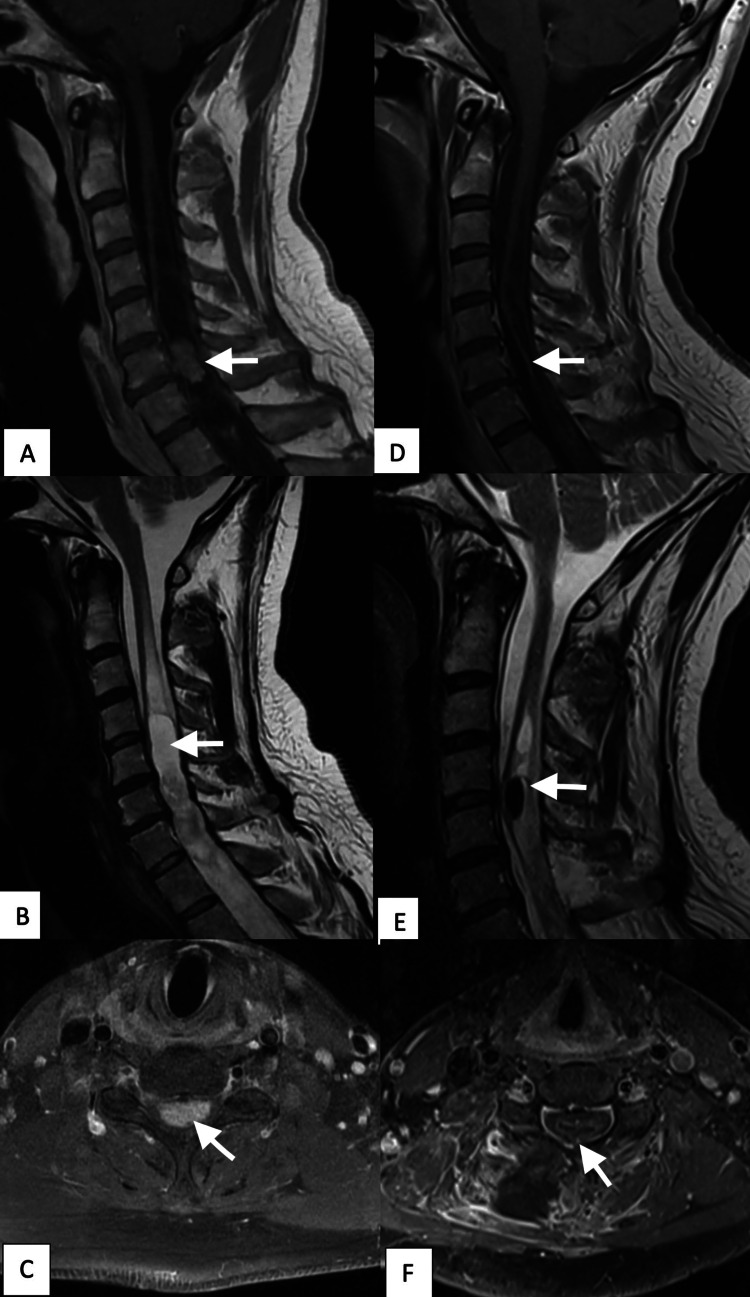
Preoperative and postoperative cervical MRI. (A) Preoperative T1-weighted MRI with Gadolinium injection showing enhancing intramedullary tumor at C6 level (arrow). (B) Preoperative T2-weighted sagittal MRI showing the syrinx (arrow). (C) Preoperative T1-weighted axial MRI with Gadolinium injection showing the tumor (arrow). (D) Post-operative T1-weighted sagittal MRI with Gadolinium injection showing complete resection of the tumor (arrow). (E) Post-operative T2-weighted sagittal MRI showing the regression of syrinx (arrow). (F) Post-operative T1-weighted axial MRI with Gadolinium injection showing complete resection of the tumor (arrow).

A complete spinal magnetic resonance imaging (MRI) with gadolinium revealed a 2.0 cm contrast enhancing intramedullary lesion at the C6-7 level, associated with a syrinx extending from C2 to T12. Its exact dimensions were 10mm height x 20mm length x 20mm width, for a total tumor size of 2,000mm^3 ^(Figure 1).

Further cerebral imaging did not reveal any intracranial lesion. We opted for surgical treatment to obtain a histologic diagnosis and to prevent clinical worsening due to the presence of the extensive syrinx.

Surgical technique

**Figure 2:**
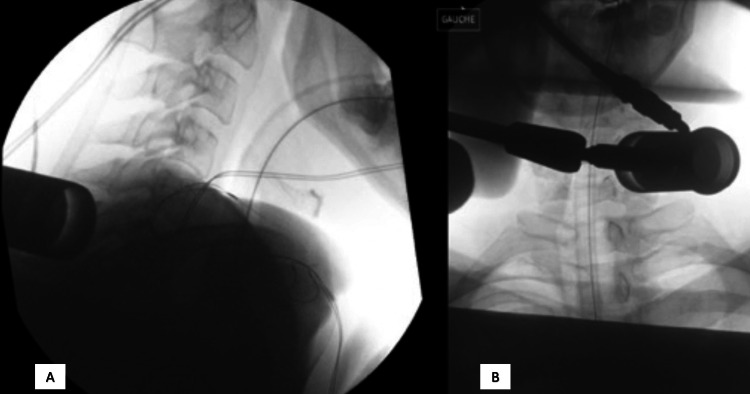
Intraoperative fluoroscopy confirming the final position of the tubular retractor. (A) Lateral view. (B) Antero-posterior view

Under general anesthesia, the patient was placed prone on a standard surgical bed, on soft cushions. Intraoperative neuromonitoring was performed, with leads for motor and somatosensory evoked potentials placed accordingly. The head was secured in a 3-points rigid cranial fixation head clamp. The surgical tape was used to retract the shoulders. Using standard 2D fluoroscopy, an 18mm incision was marked 1.5cm paramedian on the right at the C6-C7 level. Using sequential dilators, a unilateral, standard blunt paraspinal muscles splitting approach was performed, and a final 18mm by 5cm deep fixed tubular retractor (METRx, Medtronic, Minneapolis, MN, USA) was placed. Its final position was confirmed by fluoroscopy (Figure 2).

**Figure 3:**
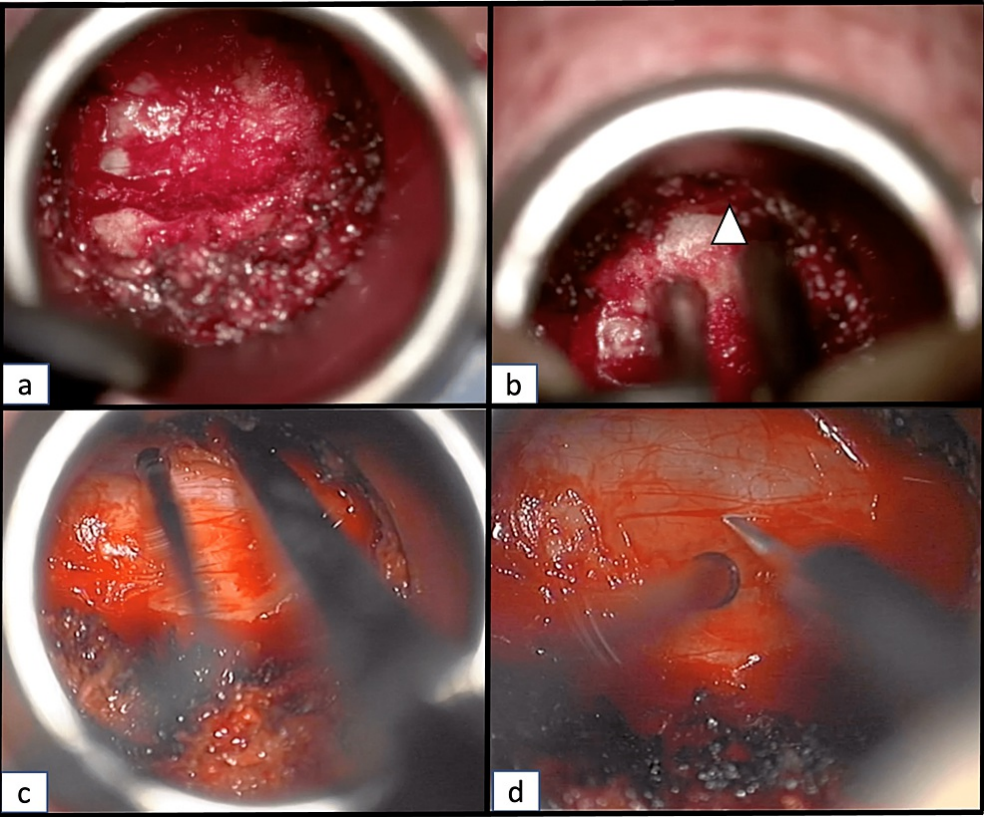
Bilateral exposure of the dura and durotomy (a) Exposure of the dura after drilling of the ipsilateral C6 hemilamina; (b) Drilling of the ventral aspect of the C6 spinous process (arrowhead) and the contralateral lamina; (c) Exposure of the contralateral edge of the dura; (d) Midline durotomy.

**Figure 4:**
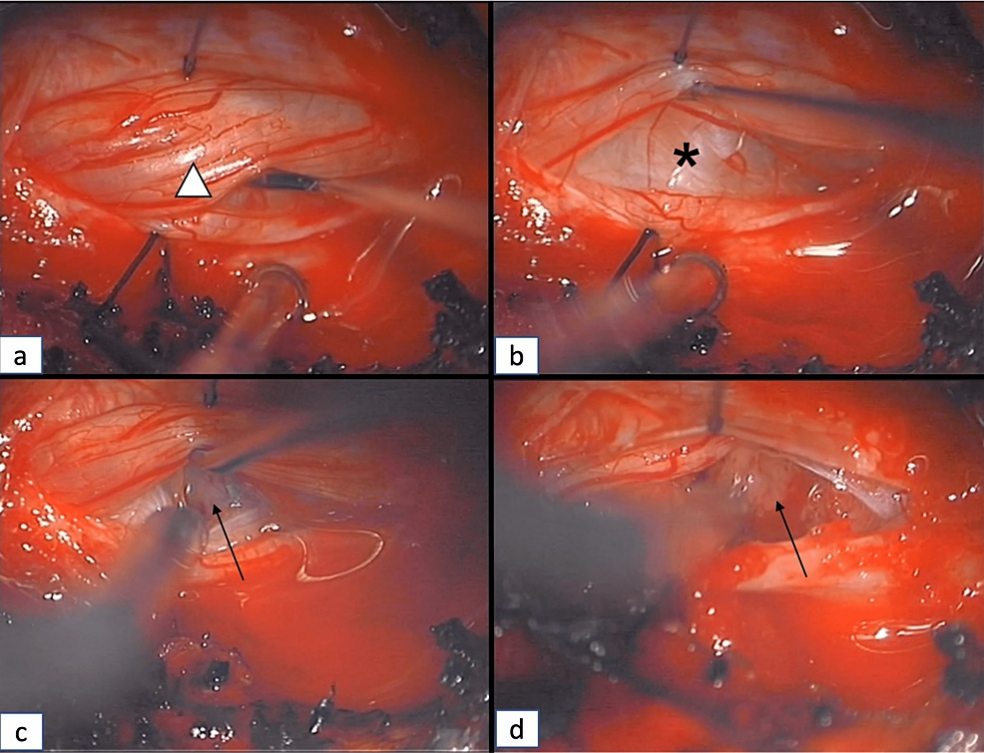
Exposure of the tumor and myelotomy (a) Nerve root (arrowhead) retraction; (b) Identification of the cyst (*) abutting the surface of the spinal cord; (c) Myelotomy at the dorsal root entry zone and identification of the tumor (arrow); (d) Exposure of the tumor (arrow) superior and inferior edges.

**Figure 5:**
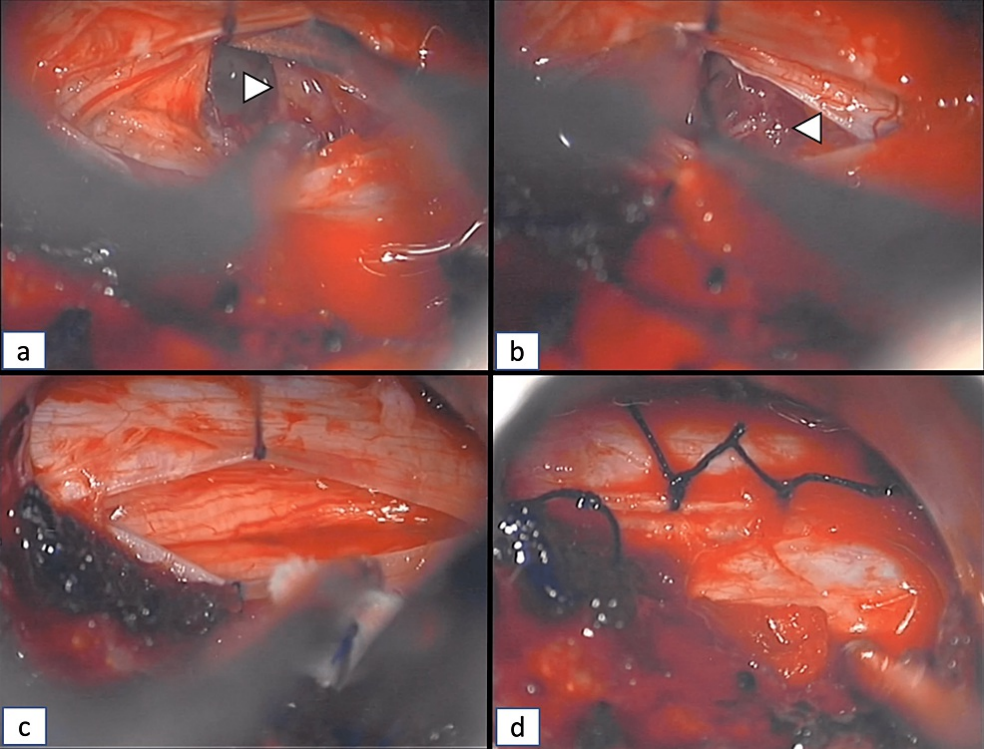
Microsurgical dissection and resection of the tumor (a) Dissection of the superior edge (arrowhead) of the tumor; (b) Dissection of the inferior edge (arrowhead) of the tumor; (c) Gross total resection of the tumor; (d) Dural closure using interrupted sutures.

Under direct neurosurgical microscope magnification and illumination, a right-sided unilateral complete C6 hemilaminectomy was performed using a high-speed Midas drill (Figure 3a). The ventral aspect of the spinous process as well as that of the contralateral lamina was drilled to allow a wide exposure of the dura mater (Figure 3b-3c). A midline durotomy was performed with an ophthalmic surgical blade (Figure 3d), and the dural leaflets were retracted with 4.0 Nurolon sutures (Ethicon-Johnson and Johnson, Bridgewater, NJ, USA). Initially, the arachnoid membrane was preserved, but was later opened with sharp bayonetted microscissors. The rostral and caudal ends of the tumor-spinal cord interface were identified. Since the cystic component of the lesion was abutting the posterior surface of the spinal cord, it was easily identified after dural opening (Figure 4a-4b). A right-sided dorsal root entry zone myelotomy was performed at the C6 level (Figure 4c). This allowed easy access to the lesion, which was well encapsulated, with a clear tumor-spinal cord interface (Figure 4d). Microsurgical dissection techniques using Rhoton micro-instruments and bayonetted microinstruments were used to free the superior, inferior, and lateral edges of the tumor (Figure 5a-5b). Since the tumor was easily mobilized due to the presence of syrinx, and with the absence of identified anterior vascular supply, we attempted to remove the lesion en-bloc. However, the lesion came out in two pieces. A gross total resection was achieved (Figure 5c). Meticulous hemostasis was maintained with the Malis bipolar and a gelatin-thrombin matrix sealant (Floseal, Baxter, Deerfield, IL, USA).

The dura was closed in the usual manner as previously described [8], using 4.0 interrupted Nurolon sutures (Figure 5d). A fibrin sealant (Tisseel; Baxter, Deerfield, IL, USA) and oxidized regenerated cellulose patch (Surgicel; Ethicon-Johnson and Johnson, Bridgewater, NJ, USA) were placed afterwards. The fascia and muscle layers were closed in a watertight manner.

Video 1 describes the surgical technique used for the resection of this intramedullary tumor.

**Video 1:** Operative technique for the resection of an intramedullary tumor of the cervical spine using minimally invasive approach with fixed tubular retractor.

## Discussion

Surgical resection of IMSC tumors is often indicated to alleviate the progression of neurological symptoms, obtain a histological diagnosis, and guide the treatment strategy. It is associated with better prognosis and survival rates [1,2].

Typically, a posterior approach with midline myelotomy or through the dorsal root entry zone for the resection of IMSC tumors, such as ependymomas and astrocytomas, is the favored route, as this helps in minimizing the mobilization of the posterior dorsal columns. Locating the exact midline is crucial in performing a safe surgery, but may be difficult, especially in the setting of uncharacteristic anatomy due to cord swelling, rotation, neovascularization, or an associated syringomyelia. In this setting, neuro-stimulation of sensory tracts may be used.

Our goal is to demonstrate that the DREZ allows for a GTR resection of the cervical intramedullary lesion, even with a minimally invasive surgical approach. For lateral IMSC tumors, the dorsal root entry zone (DREZ) is the preferred approach. The main advantage of the DREZ approach is to preserve sensory functions on the contralateral side by avoiding manipulation of the contralateral dorsal column.

The most frequently encountered long-term complication after surgery for spinal tumors is spinal kyphotic deformity [5]. In adults, a new onset of deformity or a worsening of a preoperative deformity was found in 20 to 30% of cases of open laminectomy for removal of intradural tumors [11,12]. Tumors at the cervicothoracic junction present a unique biomechanical challenge with an increased risk of instability and deformity associated with simple laminectomy, requiring instrumentation [13]. Our short-term six months follow-up demonstrates that these MIS approaches are not associated with any kyphotic deformity that is typically associated with a complete bilateral laminectomy.

In this setting, a unilateral MIS approach can present an alternative to open laminectomy and instrumentation, open laminoplasty and mini-open unilateral hemilaminectomies. When compared to open surgery for treatment of intradural-extramedullary tumors, MIS approaches are associated with shorter length of hospital stay, lower rates of narcotics use and pain postoperatively, lower blood loss and decreased rates of symptomatic CSF leak, all while achieving similar GTR rates [10,14,15].

There are few reports of MIS resection of intramedullary tumors. Yüce et al. [1] described a midline open approach, with an open unilateral subperiosteal muscle dissection and hemilaminectomy at the cervical and thoracic levels. This unilateral surgical approach still allowed for a bilateral exposure of the thecal sac. A GTR was achieved in 92.9% of cases, with a good neurological outcome at six months follow-up. Although the results of this modified approach are encouraging and present a large series (168 patients), the technique used differs from the one described in this report. The paraspinal muscles were stripped from the midline bony attachments and involved resection of at least two complete laminas.

Kruger et al. [16] reported a case series of spinal hemangioblastomas in both the cervical and thoracic level, with either a fixed nonexpendable tubular retractor or expandable retractor system. The exact type of retractor used in the resection of their cervical lesion is not well specified. As well, their largest lesion in the intramedullary cervical level was 705.8 mm^3^. In our case, it was 2,000mm^3^.

Ogden and Fessler [17] also reported a similar muscle preserving unilateral approach using an expandable tubular retractor with retracting blades for the resection of an intramedullary ependymoma. The tumor was infracentimetric, and at the thoracic (T4) level. A T4 and T5 hemilaminectomy was performed and the tumor debulked in a piecemeal fashion. They reported a good outcome, with no post-operative complications. There was less postoperative pain compared to other patients with similar pathology operated using an open approach. The patient was off narcotics on day 2 postoperatively and was cleared for hospital discharge on day 3. At the six-month follow-up, an MRI revealed no residual tumor.

In our case, we found comparable results with complete resolution of his neck pain on postoperative day 1, a 4-day hospital stay, no CSF leak or pseudomeningocele. More importantly, we were able to achieve a GTR based on postoperative imaging. Furthermore, scoliosis films at six months postoperatively did not show any kyphosis at the operated level.

Despite the relatively large tumor size of 2 cm or 2,000mm^3^, a single-level hemilaminectomy was done to completely remove the tumor, limiting the extent of ligamentous damage and bony resection. Although we recognize that tumor size may cause one to shy away from this MIS approach, we demonstrate here that such approach should not be limited to only infracentimetric intramedullary tumors. To our knowledge, we present the resection of the largest tumor size at the cervical level using a minimally invasive approach.

We recognize that the presence of the cyst in the case reported by Ogden and Fessler [17] helped in creating a cleavage interface between the tumor and the cord, isolating the dissection planes upon the myelotomy. Similarly, the presence of a syrinx facilitated in our case the microdissection and the complete resection of the IMSC lesion and was one of the criteria in the selection of the MIS approach of this IMSC tumor.

Indication considerations

MIS approach to IMSC tumors is feasible, but cases must be carefully selected. More ventral or lateral lesions are better approached through the DREZ. The presence of cyst or syrinx can greatly help tumor dissection and resection. Vascular anatomy must be meticulously assessed to identify the midline, and to rule out the presence of anterior feeders that must be addressed before complete resection of the tumor and can be a relative contraindication to an attempt of en-bloc resection of the tumor. Tumor size remains the main limitation of the use of an MIS approach, especially in the absence of a large syrinx or cyst.

Tumors that are large, firm, without adjacent cyst or syrinx or presenting a rich vascular supply especially anteriorly that is difficult to control may warrant a wider exposition not possible with MIS approaches. The main goal of surgery remains to achieve total gross resection while preserving neurological function, and not the size of the approach.

## Conclusions

We present a clinical case of complete resection of a large cervical intramedullary ependymoma via a dorsal root entry zone, using a minimally invasive approach with a fixed tubular retractor. At six months follow-up, there was an improvement in his neurological status. There was no surgical complication, nor any post-operative kyphosis reported. The post-operative pain resolved.

Although resection of the intramedullary tumor remains challenging, we demonstrate that with careful patient selection and surgical planning, a complete surgical resection of large cervical IMSC via a dorsal root entry zone approach is a safe and feasible option even using minimally invasive techniques.
